# Prognostic value of nutritional assessment tools in patients with sepsis: a systematic review with meta-analysis of PNI and GNRI

**DOI:** 10.3389/fnut.2026.1890738

**Published:** 2026-08-26

**Authors:** Zanzan Sun, Jiajing Liu, Liqun Zhu, Su Gu, Jianguo Zhang, Zhimin Tao

**Affiliations:** 1 Department of Emergency Medicine, Affiliated Hospital of Jiangsu University, Zhenjiang, Jiangsu, China; 2 School of Medicine, Jiangsu University, Zhenjiang, Jiangsu, China; 3 Department of Nursing, Affiliated Hospital of Jiangsu University, Zhenjiang, Jiangsu, China; 4 Department of Emergency Medicine, The First People's Hospital of Yancheng, Affiliated Hospital of Nanjing University Medical School, Yancheng, Jiangsu, China

**Keywords:** intensive care, meta-analysis, nutritional assessment tools, prognosis, sepsis

## Abstract

**Background:**

Malnutrition is common in patients with sepsis and is associated with prolonged hospitalization and increased mortality. Early assessment of nutritional status using standardized nutritional assessment tools may be important for risk stratification and outcome prediction.

**Methods:**

We searched PubMed, Embase, Web of Science, and the Cochrane Library from database inception to November 2025. Two reviewers independently performed study selection, data extraction, and risk-of-bias assessment, with disagreements resolved through discussion. Cohort and case-control studies evaluating nutritional assessment tools in patients with sepsis and reporting mortality-related outcomes were included.

**Results:**

A total of 11 studies involving 34,810 patients were included. All included studies had a Newcastle-Ottawa Scale (NOS) score of ≥7. Seven nutritional assessment tools were evaluated: the Prognostic Nutritional Index (PNI), Nutrition Risk in the Critically Ill (NUTRIC) score, modified NUTRIC (mNUTRIC) score, Controlling Nutritional Status (CONUT) score, Geriatric Nutritional Risk Index (GNRI), Nutritional Risk Screening 2002 (NRS 2002), and blood urea nitrogen-to-albumin ratio (BAR). Quantitative synthesis was feasible only for PNI and GNRI because of the limited number and heterogeneity of studies evaluating the other tools. A random-effects meta-analysis of studies evaluating PNI showed a pooled hazard ratio (HR) of 0.57 (95% CI: 0.51–0.64), indicating that higher PNI scores were associated with lower short-term mortality in patients with sepsis. The remaining tools were therefore summarized narratively.

**Conclusions:**

Among the nutritional assessment tools included, the NUTRIC score appeared to have favorable predictive performance and may be one of the more promising tools based on currently available evidence. Early nutritional assessment may help identify patients at increased risk of poor outcomes and support clinical risk stratification. However, the evidence remains limited because most studies were retrospective and single-center, and direct quantitative comparisons among all nutritional assessment tools were not possible. The included observational studies do not demonstrate that use of these tools or subsequent nutritional interventions improves patient outcomes. Further prospective, multicenter studies are required to validate their prognostic utility in sepsis research.

## 1 Introduction

Sepsis is one of the most common critical illnesses in the intensive care units (ICUs) and is characterized by life-threatening organ dysfunction resulting from a dysregulated host response to infection (1). It remains associated with substantial morbidity and mortality and represents a major global public health challenge (2). Through inflammatory stress, gastrointestinal disorder, and metabolic abnormality, sepsis substantially increases the risk of malnutrition (3). Therefore, malnutrition is frequent in patients with sepsis and is associated with more systemic complications, longer hospital stays, and higher risk of death (4, 5).

Nutritional status reflects physiological reserve and immune competence and may influence recovery in critically ill patients with sepsis. Early identification of nutritional risk has therefore become an important component of intensive care (3, 6–8). However, conventional indicators such as weight loss, intake reduction, and serum albumin decline can be substantially influenced by non-nutritional factors, including fluid overload, systemic inflammation, and altered hepatic metabolism, thereby limiting their reliability in clinical practice (8–10). In particular, serum albumin concentration is commonly incorporated into nutritional assessment indices; however, in patients with sepsis, it is strongly influenced by the acute inflammatory response and disease severity. Thus, albumin-based indices should be interpreted as composite markers of nutritional reserve and systemic illness rather than as specific measures of malnutrition.

Consequently, several composite assessment tools, including the Prognostic Nutritional Index (PNI), the Nutritional Risk in Critical Illness (NUTRIC), the Modified NUTRIC (mNUTRIC), and the Controlling Nutritional Status (CONUT), have been developed to integrate nutritional, immunological and inflammatory states of patients, with reported prognostic values across a variety of critically ill populations (4, 5, 8, 11, 12). Although several studies have evaluated nutritional assessment tools in patients with sepsis, the available evidence remains heterogeneous and has largely been derived from observational cohorts. For this reason, the prognostic utility of different tools, and the thresholds most relevant to key outcomes such as short-term mortality, remain uncertain. Herein we performed a systematic review and meta-analysis to identify and evaluate the available evidence on nutritional assessment tools in adult patients with sepsis, with an aim to clarify their association with mortality-related outcomes and inform early risk stratification in clinical practice.

## 2 Methods

This study of systematic review and meta-analysis was conducted and reported in accordance with the PRISMA 2020 statement (13) and was registered in PROSPERO (CRD420251185606).

### 2.1 Search strategy

PubMed, Embase, Web of Science, and the Cochrane Library (CENTRAL) were searched from database inception to November 26, 2025. The search strategy combined controlled vocabulary terms and free-text terms related to sepsis and nutritional assessment tools, including PNI, NUTRIC, mNUTRIC, CONUT, Nutrition Risk Screening 2002 (NRS-2002), Geriatric Nutritional Risk Index (GNRI), and blood urea nitrogen-to-albumin ratio (BAR). Reference lists of included studies were also screened to identify additional eligible reports. No language or date restrictions were applied. The full search strategies are provided in Supplementary Tables S2–5.

### 2.2 Inclusion and exclusion criteria

Studies were eligible for inclusion if they met all of the following criteria: (1) observational studies, including cohort or case-control studies; (2) adult patients aged ≥18 years who were diagnosed with sepsis and admitted to ICU, with an ICU stay of more than 24 h; (3) studies evaluating the association between nutritional assessment indicators or nutritional risk scores and prognosis in patients with sepsis; (4) studies reporting the cut-off value or categorical definition of the nutritional assessment indicator; (5) studies providing hazard ratios (HRs), odds ratios (ORs), or relative risks (RRs) with corresponding 95% confidence intervals (CIs), or sufficient data to calculate these estimates; and (6) studies reporting eligible mortality outcomes, including ICU mortality, in-hospital mortality, and mortality assessed at different follow-up times. For presentation and interpretation, ICU, in-hospital, and 14- to 30-day mortality were classified as short-term outcomes; 90-day mortality was considered a medium-term outcome; and mortality beyond 90 days was considered a long-term outcome. Studies were excluded if they met any of the following criteria: (1) studies without available albumin and lymphocyte count data within the first 24 h after ICU admission, when these variables were required for the nutritional assessment index; (2) duplicate publications or overlapping populations, in which case the most complete or most recent study was retained; (3) reviews, editorials, letters, case reports, study protocols, conference abstracts, or articles without full-text availability; (4) *in vitro* studies, animal experiments, or basic research studies; and (5) studies with insufficient data for effect-size extraction or calculation.

### 2.3 Data extraction and quality assessment

Two reviewers (Z.S. and J.L.) independently extracted data from the included studies using a pre-defined data extraction form. Any discrepancies were resolved through discussion, and if necessary, by consultation with a third reviewer (L.Z.). The following information was extracted: first author, publication year, country or region, study design, sample size, sex distribution, patient age, APACHE II score, SOFA score, nutritional assessment tool or index, prognostic outcome, cut-off value for the nutritional assessment indicator, effect estimates including HRs or ORs with 95% CIs and area under the receiver operating characteristic curve (AUC). The methodological quality of the included studies was assessed using the Newcastle-Ottawa Scale (NOS)(14). The NOS evaluates selection of study groups, comparability among groups, and outcome ascertainment. The total score ranges from 0 to 9 points, with studies scoring ≥7 considered to have relatively high methodological quality (15). However, NOS scores were not interpreted as indicating high certainty of evidence, because the scale does not fully account for residual confounding, selective reporting, or the limitations inherent in retrospective observational studies.

### 2.4 Statistical analysis

Meta-analyses were performed using Review Manager version 5.4 (RevMan 5.4; The Cochrane Collaboration, Cochrane Nordic Center, Copenhagen, Denmark). Data extraction was conducted independently by two authors. Between-study heterogeneity was assessed using Cochran's Q test and the *I*^2^ statistic. A random-effects model was applied when substantial heterogeneity was detected, defined as *p* < 0.10 for the Q test and/or *I*^2^ > 50%; otherwise, a fixed-effects model was used (16). Mortality outcomes were grouped according to their follow-up periods before quantitative synthesis. ICU, in-hospital, and 14- to 30-day mortality were considered short-term outcomes, whereas 90-day mortality was analyzed separately as a medium-term outcome. Outcomes beyond 90 days were treated as long-term outcomes and were not pooled with short- or medium-term mortality. Quantitative pooling was performed only when the nutritional assessment tool, effect measure, and outcome period were considered sufficiently comparable. HRs with corresponding 95% CIs were pooled to evaluate the association between nutritional assessment tools and mortality in patients with sepsis. When both univariable and multivariable estimates were available, HRs adjusted in multivariable analyses were preferentially extracted. Because the cut-off values for the PNI varied across studies and were usually derived from study-specific populations using methods such as receiver operating characteristic (ROC) curve analysis, patients were classified into high-risk and low-risk groups according to the cut-off value reported in each individual study. For studies using different reference categories, HRs and their corresponding 95% CIs were inverted to ensure consistency in the direction of effect across studies. Pooled estimates were then calculated using the inverse-variance method (17). Because only a limited number of studies evaluated the same nutritional assessment tool using sufficiently comparable effect estimates and mortality endpoints, quantitative meta-analysis was performed only for PNI and GNRI. The findings for NUTRIC, mNUTRIC, CONUT, NRS 2002, and BAR were summarized narratively. Therefore, no formal pooled comparison of predictive performance among all seven tools was conducted. Formal assessment of publication bias and subgroup analyses were not performed due to limited statistical meaningfulness.

## 3 Results

### 3.1 Literature search and characteristics of included studies

**Figure 1:**
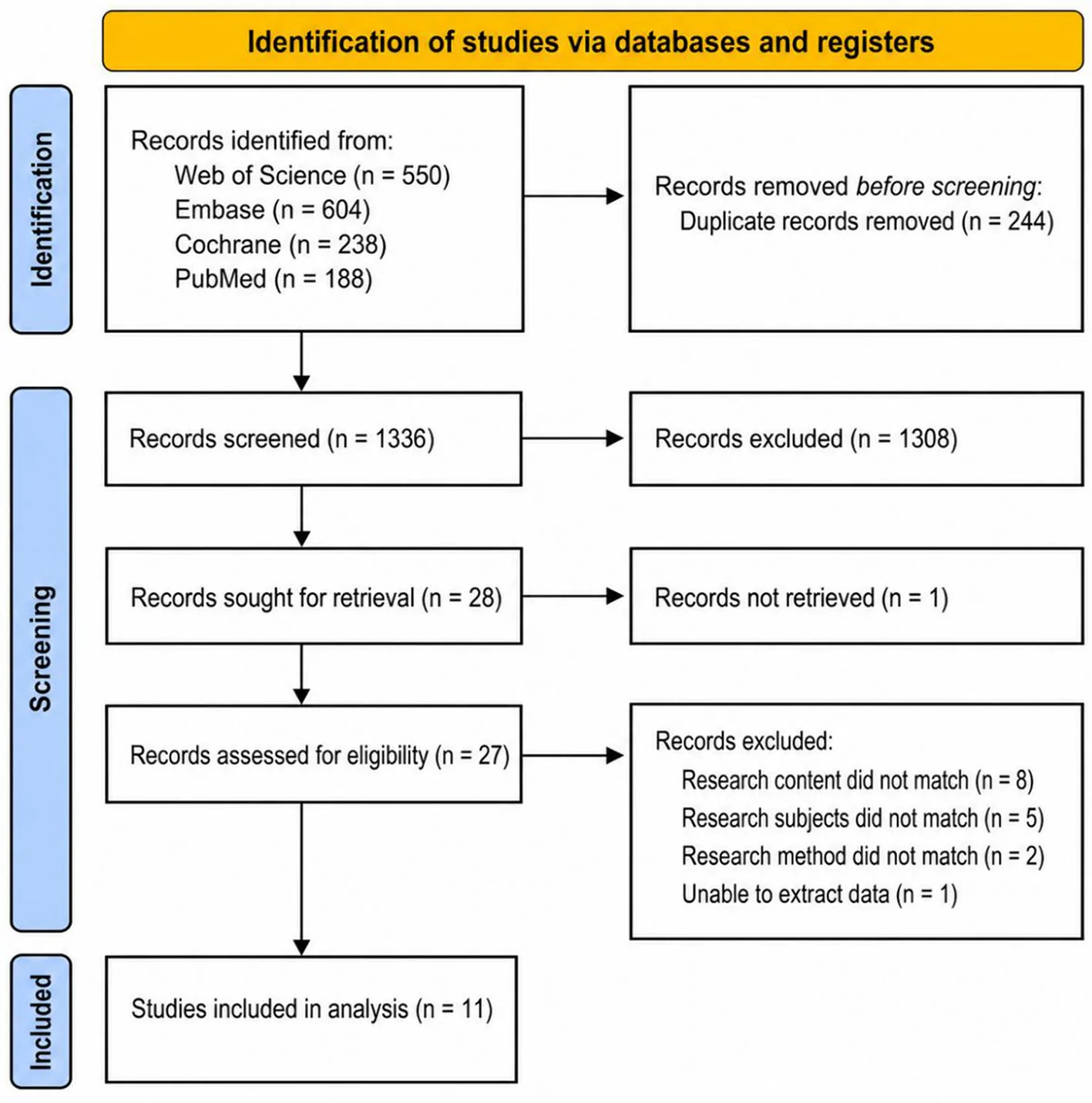
The flow diagram illustrates the literature search process.

A total of 1,580 records were identified through database searches, including Web of Science (*n* = 550), Embase (*n* = 604), Cochrane Library (*n* = 238), and PubMed (*n* = 188). After removal of 244 duplicate records, 1,336 records were then screened by title and abstract, of which 1,308 were excluded. Next, 28 articles were sought for full-text retrieval, but one article could not be obtained. Therefore, 27 full-text articles were assessed for study eligibility. Of these, 16 were excluded because of mismatched research content (*n* = 8), inappropriate study populations (*n* = 5), unsuitable research methods or study designs (*n* = 2), or unextractable data (*n* = 1). Finally, 11 studies were included in the meta-analysis. This selection process is shown in Figure 1.

The eleven eligible cohort studies included a total of 34,810 adult patients with sepsis (4–6, 10, 12, 18–23). Among these studies, two were prospective cohort studies (5, 21), whereas the remaining nine were retrospective (4, 6, 10, 12, 18–20, 22, 23). The included studies were conducted in the United States (10, 12, 20, 22, 23), China (5, 21), South Korea (4, 18), Turkey (19), and Poland (6). Most were single-center studies (4–6, 10, 12, 19–23), and only one was multicenter (18). All studies were published in English. Most studies were funded by governmental organizations (5, 10, 21–23) or academic institutions (6, 20) with three studies receiving no funding (4, 18, 19) and one study not disclosing funding sources (12).

The reported mean or median age of patients included was generally above 60 years, reflecting the pre-dominance of older adults among critically ill patients with sepsis. Disease severity was commonly evaluated using APACHE II and SOFA scores. In studies reporting APACHE II scores, most cohorts had median or mean values of approximately 20 or higher, indicating substantial illness severity. Reported SOFA scores ranged from 3 to 10, suggesting varying degrees of sepsis-related organ dysfunction across the included patients. In addition, hypertension, diabetes, heart disease, and kidney disease (including chronic kidney disease and acute kidney injury) were common comorbidities in patients with sepsis.

Moreover, the included studies evaluated several nutritional assessment tools for their prognostic value in patients with sepsis. Four studies assessed the PNI (12, 18, 19, 22), four assessed the NUTRIC (4, 5, 19, 21), three assessed the GNRI (10, 19, 20), two assessed the mNUTRIC (4, 6), two assessed the CONUT (18, 19), two assessed the NRS-2002 (10, 21), and one assessed the BAR (23). Among them, some studies evaluated more than one nutritional assessment tool.

Mortality endpoints varied across the included studies, including ICU mortality (2 studies (5, 10)), 14-day mortality (1 study (22)), 28-day mortality (6 studies (4, 6, 10, 20–22)), 30-day mortality (3 studies (12, 18, 23)), 90-day mortality (2 studies (12, 22)), and 360-day mortality (1 study (23)). More detailed characteristics are available in Table 1. For clarity, ICU, in-hospital, and 14- to 30-day mortality were summarized as short-term outcomes, whereas 90-day and 360-day mortality were reported as medium- and long-term outcomes, respectively. These outcomes were not considered clinically interchangeable.

**Table 1:** Baseline characteristics and quality assessment of the included studies evaluating nutritional assessment tools in patients with sepsis.

No.	Study	Size	Country	Design	Sepsis definition	Age (year)	APACHE II score	SOFA score	NATs	Cut-off value	Total NOS
1	Wu et al. (12)	2669	US	R	Sepsis-3: suspected infection plus SOFA increase ≥2	66.2 ± 16.5	47.3 ± 16.4	7.4 ± 4.0	PNI	29.3	8
2	Pan et al. (22)	6,234	US	R	Sepsis-3: suspected infection plus SOFA ≥2	66.0 (18.0–90.0)	20.0 (1.0–53.0)	7.0 (0.0–23.0)	PNI	—	8
3	Calili et al. (19)	126	Turkey	R	Sepsis-3 criteria	73.0 ± 14.9	28.1 ± 8.1	8.5 ± 2.9	NRS 2002/NUTRIC/ CONUT/PNI/GNRI	NUTRIC: 6	7
4	Baek et al. (18)	9,763	SK	R	Sepsis-3: infection plus SOFA ≥2	75 (62–84)	17 (13–22)	5 (3–7)	PNI/CONUT	—	8
5	Jeong et al. (4)	482	SK	R	Institutional ASAN Sepsis Cohort; exact consensus criteria not clearly reported	66 (56–74)	21 (16–28)	10 (7–14)	NUTRIC/mNUTRIC	6	8
6	Wełna et al. (6)	146	Poland	R	Sepsis-3: infection plus acute SOFA increase ≥2	66 (58–77)	21 (15–27)	10 (7–13)	mNUTRIC	6	8
7	Xie et al. (5)	245	China	*P*	Sepsis-3: infection plus SOFA increase ≥2	61.3 ± 16.1	23.5 ± 7.8	6.7 ± 3.6	NUTRIC	6	9
8	Wang et al. (21)	140	China	*P*	Sepsis-3 with AKI defined by KDIGO	83 (73–88)	—	—	NUTRIC/NRS 2002	NUTRIC: 6/NRS 2002: 5	8
9	Li et al. (20)	2,834	US	R	Sepsis-3: suspected infection plus SOFA increase ≥2	76.8 (70.5–83.4)	—	3 (2–5)	GNRI	—	8
10	Cai et al. (10)	4,515	US	R	Sepsis-3 with AKI defined by KDIGO	76.0 (70.0, 83.0)	—	3.0 (2.0–5.0)	GNRI	—	9
11	Wang et al. (23)	7,656	US	R	Sepsis-3: suspected infection plus SOFA increase ≥2	64.0 (53.0, 76.0)	—	7.0 (4.0–10.0)	BAR	—	9

R, retrospective; P, prospective; APACHE II, acute physiology and chronic health evaluation II; SOFA, sequential organ failure assessment; NATs, nutritional assessment tools; NOS, Newcastle-Ottawa scale; US, the United States; SK, South Korea.

### 3.2 The prognostic values of nutritional assessment tools on the clinical outcome of patients with sepsis

#### 3.2.1 PNI

**Figure 2:**
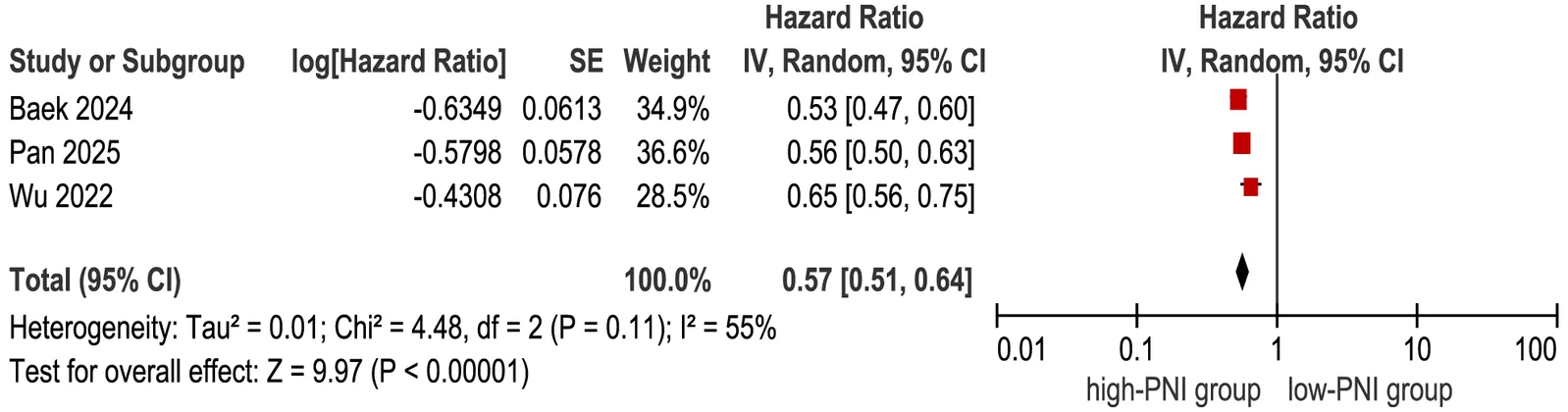
Forest plot of the association between PNI and short-term mortality in patients with sepsis. Effect estimates are presented as hazard ratios with 95% confidence intervals.

**Figure 3:**
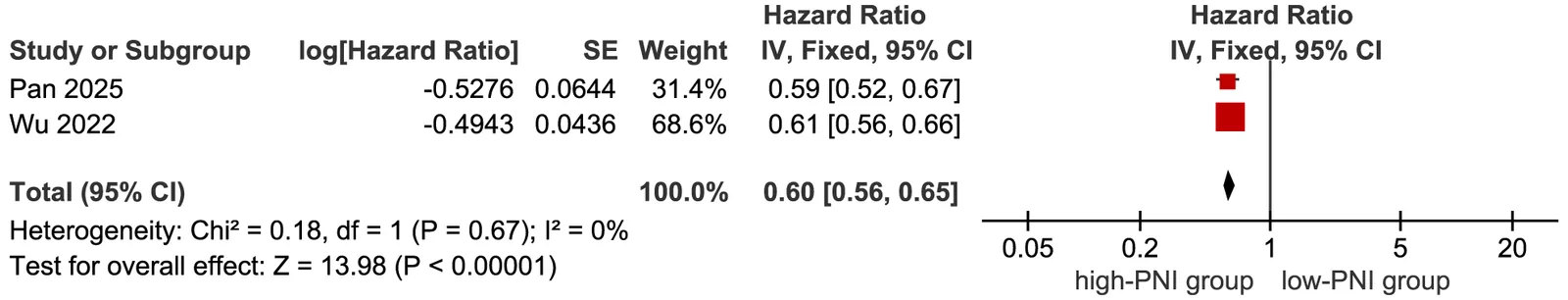
Forest plot of the association between PNI and 90-day mortality in patients with sepsis. Effect estimates are presented as hazard ratios with 95% confidence intervals.

A total of four studies evaluated the association between PNI and mortality risk in patients with sepsis. Among them, three studies reported HRs with 95% CIs for the association between PNI and 14–30-day mortality (12, 18, 22), whereas one study assessed in-ICU mortality (19). For short-term mortality, three studies were included in the meta-analysis. Moderate heterogeneity was observed among the studies (*p* = 0.11, *I*^2^ = 55%); therefore, a random-effects model was applied. The pooled HR was 0.57 (95% CI: 0.51–0.64), indicating that a higher PNI score was associated with a lower risk of short-term mortality in patients with sepsis (Figure 2). One study further examined the association between PNI and ICU mortality (22). In that study, PNI scores were significantly lower in non-survivors than in survivors. Univariate logistic regression showed that lower PNI was associated with increased mortality risk; however, this association was no longer statistically significant after adjustment in multivariable analysis, suggesting that PNI may reflect overall disease severity or nutritional status rather than serve as an independent predictor of ICU mortality. The ICU mortality was not pooled with these fixed-time outcomes and was summarized narratively. Similarly, for 90-day mortality, the pooled HR was 0.60 (95% CI: 0.56–0.65), suggesting a consistent protective association between higher PNI and reduced medium-term mortality risk (Figure 3).

In addition, non-linear associations between PNI and all-cause mortality on 14, 28, and 90 days were reported. The ROC analysis and a two-stage Cox proportional hazards model identified threshold effects for PNI at each time point. Below these inflection points, each 1-unit increase in PNI was associated with an approximately 9% reduction in mortality risk. However, above these thresholds, the association appeared to reverse, with higher PNI values linked to increased mortality risk. This finding suggests that the relationship between PNI and mortality may not be strictly linear and should be interpreted with caution.

Regarding the predictive performance of PNI for short-term mortality in patients with sepsis, Wu et al. (12) reported an area under the receiver operating characteristic curve (AUC) of 0.6436 for PNI (95% CI: 0.6204–0.6625). This value was lower than that of the Sequential Organ Failure Assessment (SOFA) score (AUC: 0.6933, 95% CI: 0.6708–0.7158), but significantly higher than those of the neutrophil-to-lymphocyte ratio (NLR; AUC: 0.5962, 95% CI: 0.5717–0.6206; *p* = 0.0031), red cell distribution width (RDW; AUC: 0.5878, 95% CI: 0.5629–0.6127; *p* < 0.0001), and platelet-to-lymphocyte ratio (PLR; AUC: 0.4979, 95% CI: 0.4722–0.5235; *p* < 0.0001). Notably, the combined model incorporating PNI and SOFA achieved a higher AUC of 0.7140 (95% CI: 0.6921–0.7360), which was significantly greater than that of SOFA alone (*p* < 0.0001). Similar findings were reported by Pan et al. (22), who showed that although PNI alone had weaker predictive performance than SOFA, the combination of PNI with SOFA improved mortality prediction compared with SOFA alone.

#### 3.2.2 NUTRIC and mNUTRIC

In the included studies, four assessed the NUTRIC score (4, 5, 19, 21), and two evaluated the mNUTRIC score (4, 6). We found that higher NUTRIC and mNUTRIC scores were consistently associated with increased mortality risk in patients with sepsis, suggesting that these tools may be useful for identifying critically ill patients at increased prognostic risk. However, these findings were derived from a limited number of heterogeneous observational studies and should therefore be interpreted cautiously. Two studies (4, 21) reported the prognostic value of the NUTRIC score for short-term, particularly 28-day, mortality in patients with sepsis. Among them, Jeong et al. (4) found that the NUTRIC score showed good discriminatory performance for 28-day mortality, with an AUC of 0.762 (95% CI: 0.718–0.806). Similarly, Wang et al. (21) compared the predictive performance of the NUTRIC score with that of the NRS 2002 score, serum albumin, and body mass index (BMI). In that study, the NUTRIC score yielded the highest AUC among the evaluated indicators at 0.785 (95% CI: 0.708–0.850).

Furthermore, two studies (5, 19) evaluated the association between NUTRIC score and ICU mortality in patients with sepsis. Xie et al. (5) reported that the NUTRIC score was positively associated with ICU mortality. After adjustment for confounding factors, each increase in NUTRIC score was associated with a heightened risk of ICU death, with an OR of 1.92 (95% CI: 1.32–2.80; *p* = 0.001). Calili et al. (19) further assessed the prognostic value of five nutritional assessment tools for ICU mortality by evaluating nutritional status both at admission and on day 5 of hospitalization. In survivors, the NUTRIC score decreased from day 0 to day 5, whereas no significant reduction was observed among non-survivors. Although several nutritional assessment tools were associated with ICU mortality in univariate logistic regression analysis, multivariable analysis showed that only a high NUTRIC score on day 5 (>6 points) was independently associated with high ICU mortality (*p* < 0.05).

Regarding the mNUTRIC score, two studies (4, 6) evaluated its predictive value for short-term, specifically 28-day, mortality in patients with sepsis. Wełna et al. (6) reported that the mNUTRIC score calculated at ICU admission had good prediction ability for 28-day mortality, with an AUC of 0.833 (95% CI: 0.76–0.89). At the optimal cutoff value of 6 points, the sensitivity and specificity were 90% and 63%, respectively. In parallel, Jeong et al. (4) found that an mNUTRIC cutoff score of 6 points provided acceptable predictive performance for 28-day mortality, with an AUC of 0.757 (95% CI: 0.713–0.801). However, its performance value was lower than that of the APACHE II score, which had an AUC of 0.826 (95% CI: 0.787–0.866). They also compared the prognostic values of original NUTRIC score with that of mNUTRIC score. The NUTRIC score had an AUC of 0.762 (95% CI: 0.718–0.806), and no statistically significant difference was found between the ROC curves of the NUTRIC and mNUTRIC scores (*p* = 0.45), indicating their comparable predictive performance for 28-day mortality in patients with sepsis (Table 2).

**Table 2:** Comparison of AUCs for NUTRIC and mNUTRIC.

Study	Tools	Outcome	Cut-off	AUC	95% CI
Wang et al. (21)	NUTRIC	28-day mortality	6	0.73	0.65–0.80
Jeong et al. (4)	NUTRIC	28-day mortality	6	0.76	0.72–0.81
	mNUTRIC	28-day mortality	6	0.76	0.71–0.80
Wełna et al. (6)	mNUTRIC	28-day mortality	6	0.83	0.76–0.89
Calili et al. (19)	NUTRIC (day 5)	ICU mortality	6	0.77	0.69–0.84
Xie et al. (5)	NUTRIC	ICU mortality	6	—	—

#### 3.2.3 GNRI

**Figure 4:**
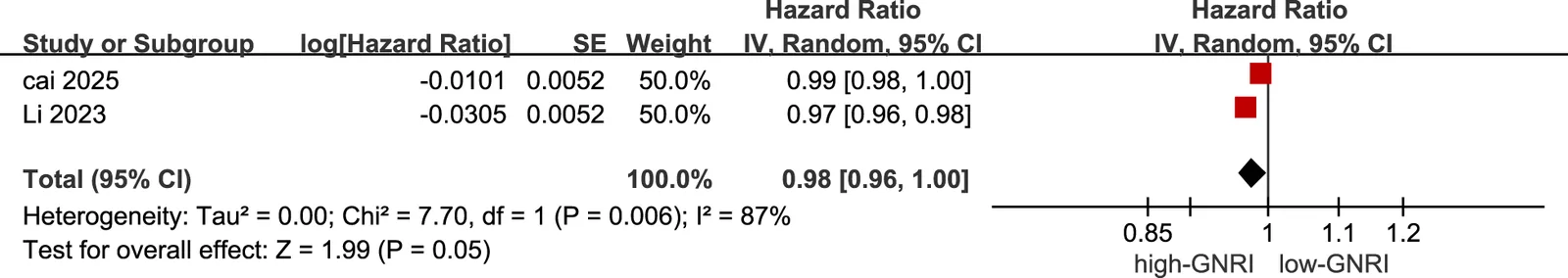
Forest plot of the association between GNRI and 28-day mortality in patients with sepsis. Effect estimates are presented as hazard ratios with 95% confidence intervals.

Three studies evaluated the association between GNRI score and mortality in patients with sepsis (10, 19, 20). Two studies (10, 20) reported that higher GNRI scores were associated with a lower risk of 28-day mortality. The pooled HR was 0.97 (95% CI: 0.96–1.00), suggesting a modest reduction in short-term mortality risk with each one-unit increase in GNRI (Figure 4). In contrast, Calili et al. (19) adopted ICU mortality as the primary outcome and found no significant association between GNRI score and mortality in patients with sepsis.

Further dose-response analyses suggested that the relationship between GNRI and mortality may be non-linear and context-dependent. Cai et al. (10) used restricted cubic spline analysis and found a monotonically decreasing association between GNRI and 28-day mortality in patients with sepsis-associated acute kidney injury (AKI), although the test for non-linearity was not statistically significant (*p* for non-linearity = 0.207). The optimal cutoff value was 83.416. By contrast, Li et al. (20) reported an L-shaped relationship between GNRI and 28-day mortality, with significant evidence of non-linearity (*p* for non-linearity = 0.021) and an inflection point at 98.1. Below this threshold, GNRI was negatively associated with 28-day mortality (HR = 0.967, 95% CI: 0.959–0.974), whereas above the threshold, no significant association was observed (HR = 1.043, 95% CI: 0.984–1.106; *p* = 0.1549).

Taken together, these findings suggest that lowered GNRI scores may be associated with increased short-term mortality in patients with sepsis, particularly among patients with sepsis-associated AKI or GNRI values below clinically relevant thresholds. However, the prognostic value of GNRI for ICU mortality remains uncertain.

#### 3.2.4 CONUT

Two studies (18, 19) evaluated the association between the CONUT score and mortality in patients with sepsis. Baek et al. (18) examined the relationship between CONUT score and 30-day mortality, concluding that the median CONUT score among patients with sepsis was 5 (IQR: 3–7). Compared with patients without malnutrition according to CONUT criteria (CONUT score: 0–1), those with moderate malnutrition (CONUT score: 5–8) or severe malnutrition (CONUT score: 9–12) had significantly higher risks of 30-day mortality. The HRs were 1.52 (95% CI: 1.24–1.87; *p* < 0.001) for moderate malnutrition and 2.42 (95% CI: 1.95–3.02; *p* < 0.001) for severe malnutrition. Calili et al. (19) assessed the relationship between CONUT score and ICU mortality and found that non-survivors had higher CONUT scores than survivors. Although univariate logistic regression indicated an association between CONUT score and ICU mortality in patients with sepsis, this association did not remain significant after adjustment in multivariable analysis. Therefore, the available findings suggest that higher CONUT scores may be associated with increased short-term mortality in patients with sepsis, although their independent prognostic value for ICU mortality remains uncertain.

#### 3.2.5 NRS 2002

Wang et al. (21) conducted the Cox regression analysis and showed that the NRS 2002 score was independently associated with mortality in patients with sepsis-associated AKI. The ROC curve analysis demonstrated that the NRS 2002 score had moderate predictive performance for mortality, with an AUC of 0.728 (95% CI: 0.647–0.800). Although this AUC was lower than that of the NUTRIC score (AUC = 0.785, 95% CI: 0.708–0.850), the difference between the two ROC curves was not statistically significant. Kaplan-Meier survival analysis further showed that patients with NRS 2002 scores ≥5 had significantly worse prognosis than those with NRS 2002 scores < 5. The 28-day cumulative survival rate was 42.1% in patients with NRS 2002 scores ≥5, compared with 75.6% in those with NRS 2002 scores < 5 (log-rank test: χ^2^ = 11.884; *p* = 0.001).

#### 3.2.6 BAR

Wang et al. (23) reported that higher BAR values at ICU admission were associated with increased mortality. In multivariable Cox regression analysis, patients in the high BAR group had a significantly higher risk of 30-day mortality than those in the low BAR group (HR = 1.219, 95% CI: 1.095–1.357; *p* < 0.001). The study also compared the prognostic performance of BAR with that of the SOFA score, blood urea nitrogen (BUN), and albumin. For 30-day mortality, the ROC curve analysis showed that BAR had an AUC of 0.661, which was slightly lower than that of the SOFA score (AUC = 0.703), but higher than that of BUN alone (AUC = 0.646) or albumin alone (AUC = 0.586). For 360-day mortality, the AUC of BAR was 0.668, higher than any of the other three indicators. Therefore, these findings suggested that BAR at ICU admission may serve as a simple and clinically accessible prognostic marker for mortality risk stratification in patients with sepsis. Nevertheless, because this evidence was derived from a single study, further validation is required.

### 3.3 Sensitivity analysis and publication bias

#### 3.3.1 Sensitivity analysis

In this study we conducted a sensitivity analysis to assess the robustness of the pooled association between the PNI and the risk of short-term mortality in patients with sepsis. A leave-one-out approach was used, in which each study included in the meta-analysis was sequentially excluded. After each exclusion, the HRs and 95% CIs from the remaining studies were re-pooled using a random-effects model. Changes in the direction, magnitude, and statistical significance of the pooled effect estimates were then examined to determine whether any single study exerted a disproportionate influence on the overall result. The sensitivity analysis showed that sequential exclusion of individual studies did not substantially alter the pooled effect estimate, its direction, or its statistical significance. This result suggested that the association between higher PNI and lower short-term mortality risk in patients with sepsis was stable and that the pooled result was not driven by any single study.

#### 3.3.2 Publication bias

Because fewer than 10 studies were included in the relevant meta-analysis, publication bias could not be reliably assessed. Therefore, funnel plots were not constructed, and formal statistical tests for publication bias, such as Egger's test or Begg's test, were not performed. This review was conducted in accordance with the Preferred Reporting Items for Systematic Reviews and Meta-Analyses (PRISMA) guidelines. To reduce the potential risk of selection and publication bias, we used a comprehensive search strategy, pre-defined inclusion and exclusion criteria, and a standardized study screening process. However, since the number of included studies was limited, the possibility of publication bias cannot be completely excluded.

## 4 Discussion

This systematic review evaluated the prognostic value of seven nutritional assessment tools in patients with sepsis. Eleven cohort studies involving 34,810 patients were included. Although all included studies scored at least 7 on the NOS, this finding should not be interpreted as evidence of high overall certainty. Quantitative meta-analysis was feasible only for PNI and GNRI, whereas the evidence for NUTRIC, mNUTRIC, CONUT, NRS 2002, and BAR was summarized narratively. Our findings summarize the prognostic associations rather than provide a direct quantitative comparison of all assessment tools. The available evidence suggests that several nutritional assessment tools may provide clinically relevant information for mortality risk stratification in patients with sepsis. But they do not demonstrate that the scores themselves influence outcomes or that interventions guided by these scores reduce mortality. The meta-analysis indicated that higher PNI scores were associated with lower short-term and medium-term mortality. Based on the available narrative evidence, the NUTRIC score appeared to have favorable predictive performance and may be one promising tool for critically ill patients with sepsis. Nevertheless, direct quantitative comparisons among all tools were not possible, and these findings should not be interpreted as establishing the superiority of one tool over the others.

Sepsis is a life-threatening condition characterized by immune dysregulation, systemic inflammation, and profound metabolic stress (24). The resulting acute catabolic response can accelerate protein breakdown, deplete energy reserves, and worsen nutritional status (25). Nutrition therapy can improve muscle atrophy, oxidative stress, and immune dysfunction, thereby compensating for the energy imbalance caused by stress and trauma (26). Accordingly, nutritional assessment has become an important component of risk stratification in patients with sepsis. Currently, a variety of nutritional screening or assessment tools have been developed, and several have been applied to identify nutritional risk and predict clinical outcomes in patients with sepsis (8).

Among these tools, the PNI is a classic and easily obtainable indicator based on serum albumin concentration and total lymphocyte count, which can be influenced by both nutritional reserve and immunoinflammatory status (27). Albumin may decrease because of acute inflammation, capillary leakage, hemodilution, and increased degradation, while lymphopenia may reflect immune dysfunction and disease severity. Therefore, a low PNI should not be interpreted as a specific marker of malnutrition alone (22). Similar caution applies to GNRI and CONUT because both indices incorporate serum albumin. Initially developed to evaluate the relationship between nutritional conditions and postoperative outcomes in surgical patients (8), PNI has subsequently shown prognostic value in malignancies and critical illnesses (28). In this meta-analysis, higher PNI scores were associated with a lower risk of short-term mortality in patients with sepsis, with a pooled HR of 0.57 (95% CI: 0.51–0.64). Our finding supports the value of PNI as a clinically relevant prognostic marker in patients with sepsis.

As for predictive performance, Kollu et al. demonstrated that PNI was independently associated with ICU mortality in patients with sepsis, although its prognostic value was lower than that of the SOFA and APACHE II scores (29). Consistently, studies included in this review reported that the AUC value of PNI alone was lower than that of the SOFA score (12, 22). However, when PNI was combined with SOFA, the predictive performance was improved to exceed that of SOFA alone. These findings suggest that integrative indicators reflecting nutritional and immunological status and organ (dys)function may enhance prognostic assessment in patients with sepsis, consistent with previous evidence that combined multi-indicator models generally outperform single-marker approaches (30). Compared with scoring systems such as APACHE II, which require more complex clinical and physiological variables, PNI is simple, inexpensive, and readily accessible from routine laboratory tests (28). Therefore, our findings here highlight the prognostic value of PNI in sepsis and support its use as a practical adjunct for mortality risk stratification and nutritional assessment in clinical practice.

Based on the currently available evidence, the NUTRIC score appears to have favorable predictive performance among the nutritional assessment tools evaluated. This observation may partly reflect the design of the NUTRIC score, which was developed specifically for critically ill patients and incorporates variables related to disease severity, including age, comorbidities, SOFA score, APACHE II score, and IL-6. These variables reflect organ dysfunction, systemic inflammation, and physiological abnormality, which may therefore contribute to its prognostic performance in patients with sepsis (4). Alternatively, the mNUTRIC score, a simplified version of the NUTRIC score, excludes IL-6 biomarker that is not routinely available in clinical settings. Despite this simplification, previous studies have shown that its predictive performance is not substantially higher than that of the NUTRIC score (4, 31, 32). Therefore, the mNUTRIC score may represent a more practical substitute in primary care settings, resource-limited hospitals, or clinical situations where inflammatory cytokine measures are unavailable. In parallel, because the evidence was derived from a small number of heterogeneous studies and no direct quantitative comparison of all tools was feasible, the apparent advantage of NUTRIC should be considered preliminary rather than definitive.

Here in the reviewed studies, the optimal cutoff value for either NUTRIC or mNUTRIC score was generally 6, as scores above this threshold were associated with increased mortality among patients with sepsis. In terms of predictive performance, the NUTRIC score achieved an AUC of 0.762–0.785 for 28-day mortality, and a higher NUTRIC score was associated with higher risk of ICU mortality with an adjusted OR of 1.92. Notably, the NUTRIC score assessed on day 5 of hospitalization was the only independent predictor of ICU mortality among the nutritional assessment tools examined (19). This finding suggests that the prognostic value of nutritional assessment in sepsis may be strengthened when evaluated dynamically rather than at a single time point.

The GNRI is another nutritional assessment tool designed primarily for older adults and is calculated using height, body weight, and serum albumin concentration (33). In our study, higher GNRI scores were associated with lower 28-day mortality in patients with sepsis, consistent with previous reports linking low GNRI to poor outcomes in heart failure (34), acute coronary syndrome (35), and chronic kidney disease (36). For patients with sepsis, Cai et al. reported an approximately monotonic inverse relationship between GNRI and 28-day mortality (10), whereas Li et al. identified an L-shaped association, with the protective effect mainly observed below the inflection point (20). The heterogeneity in the curves may stem from differences in disease severity, nutritional status, sample size, and regression adjustment among the included populations. Despite these differences, both studies support that low GNRI, reflecting malnutrition, is associated with worse prognosis. It is concluded that GNRI may be useful for prognostic risk stratification in elderly patients with sepsis.

Other nutritional assessment tools showed variable prognostic value and clinical applicability in patients with sepsis. The CONUT score can stratify patients according to the degree of malnutrition, and patients with moderate to severe malnutrition have been reported to have significantly higher 30-day mortality, in line with other report (37). However, in the multivariable analysis CONUT was not an independent predictor of ICU mortality (19). This discrepancy may be partly explained by differences in study design, patient selection, adjustment for confounders, and the limited number of outcome events. Further validation studies are needed to clarify the prognostic value of CONUT in patients with sepsis. Moreover, as a general nutritional risk screening tool, NRS-2002 shows some prognostic value in patients with sepsis-associated AKI, but its AUC is lower than that of the NUTRIC score. This indicator can be more suitable for initial nutritional screening in emergency settings (21). BAR, a novel indicator integrating renal function and protein status, demonstrates superior predictive performance compared to serum urea nitrogen or serum albumin alone. Although it provided a potential new approach for risk assessment in patients with sepsis, current evidence is based on only one study as its prognostic value requires further validation.

The strengths of this study include adherence to the PRISMA guidelines, systematic searches of multiple databases without language or date restrictions, and the inclusion of studies with NOS scores of at least 7. The included studies consisted of 34,810 patients. To our knowledge, this is the first systematic review to summarize and compare the available evidence regarding nutritional assessment tools in patients with sepsis. Meta-analyses of PNI and GNRI were also performed where sufficient data were available. The findings of this review support the potential use of nutritional assessment tools for identifying patients at increased prognostic risk. However, these associations should not be interpreted as evidence that score-guided nutritional intervention improves patient survival. Randomized or prospectively controlled studies are needed to determine whether incorporating these tools into clinical decision-making may lead to improved outcomes.

However, several limitations should be acknowledged. First, most included studies were retrospective, single-center observational studies and were therefore susceptible to selection bias, residual confounding, and variations in local clinical practice. Although all studies scored ≥7 on the NOS, overall certainty remained limited. Simultaneously, causal inferences regarding mortality or the benefits of score-guided interventions cannot be made. Second, considerable clinical and methodological heterogeneity existed in patient characteristics, sepsis definitions, mortality endpoints, covariate adjustment, and statistical methods. For example, the included studies used heterogeneous mortality endpoints, including ICU, in-hospital, 14-day, 28-day, 30-day, 90-day, and 360-day mortality. These outcomes reflect different stages of illness and recovery and may be influenced by hospital discharge and transfer practices. Although outcomes were grouped by different follow-up periods and were analyzed separately where possible, residual clinical heterogeneity remained, which might limit the comparability and generalizability of the findings. Third, cut-off values varied across studies and were often derived from study-specific ROC analyses, limiting their generalizability and preventing the establishment of standardized clinical thresholds. Fourth, quantitative synthesis was feasible only for PNI and GNRI and included few studies, whereas the remaining tools were summarized narratively; therefore, comparisons of predictive performance should be regarded as exploratory rather than definitive. Finally, the small evidence base precluded robust subgroup analyses and reliable assessment of publication bias. Larger prospective multicenter studies using standardized definitions, timing of nutritional assessment, outcomes, and externally validated cut-off values are warranted.

## 5 Conclusion

Nutritional assessment tools may provide useful prognostic information in patients with sepsis. Based on currently available evidence, the NUTRIC score appears to have favorable predictive performance and may be a promising tool for risk stratification in critically ill patients, while PNI, mNUTRIC, GNRI, CONUT, NRS 2002, and BAR may have complementary clinical applications. Among them, PNI and mNUTRIC had distinct clinical advantages. GNRI might be useful for nutritional assessment in older patients with sepsis, whereas CONUT could help identify the severity of malnutrition and inform the adjustment of nutritional support. However, direct comparisons among these tools remain limited, and no definitive conclusion regarding the superiority of a particular tool can presently be made. Early nutritional assessment, combined with clinical severity evaluation and dynamic disease monitoring, may support individualized nutritional management. But no causal relationship can be inferred, and the effectiveness of assessment-guided nutritional interventions remains unknown. Further prospective, multicenter studies using standardized cut-off values and outcome definitions are necessitated to validate these findings.
