# Supplementary material for: Prognostic value of nutritional assessment tools in patients with sepsis: a systematic review with meta-analysis of PNI and GNRI
Source: Front Nutr. 2026 Aug 26;13:1890738. doi: 10.3389/fnut.2026.1890738 (PMC13553384; doi:10.3389/fnut.2026.1890738)
Supplement: Supplementary file 1 [file Table_1.docx]

**Supplementary materials**

for

**Prognostic Value of Nutritional Assessment Tools in Patients with Sepsis: A Systematic Review and Meta-analysis**

Zanzan Sun^1^, Jiajing Liu^2^, Liqun Zhu^3^, Su Gu^4^, Jianguo Zhang^1^, Zhimin Tao^1,2^

^1^Department of Emergency Medicine, Affiliated Hospital of Jiangsu University, Zhenjiang, Jiangsu 212001, China.

^2^School of Medicine, Jiangsu University, Zhenjiang, Jiangsu 212013, China.

^3^Department of Nursing, Affiliated Hospital of Jiangsu University, Zhenjiang, Jiangsu 212001, China.

^4^Department of Emergency Medicine, The First People’s Hospital of Yancheng, Affiliated Hospital of Nanjing University Medical School, Yancheng, Jiangsu 224006, China.

^*^ Correspondences should be addressed to:

Jianguo Zhang: [1000011431@ujs.edu.cn](mailto:1000011431@ujs.edu.cn)

Zhimin Tao: [jsutao@ujs.edu.cn](mailto:jsutao@ujs.edu.cn)

## Table S1. The results of literature evaluation using the Newcastle-Ottawa Scale (NOS).

| **No.** | **Study** | **SELECTION** | | | | **COMPARABILITY** | **OUTCOME** | | | **Total** |
| --- | --- | --- | --- | --- | --- | --- | --- | --- | --- | --- |
|  |  | **1) Representative-ness of the Exposed Cohort** | **2) Selection of the Non-Exposed Cohort** | **3) Ascertainment of Exposure** | **4) Demonstration That Outcome of Interest Was Not Present at Start of Study** | **1) Comparability of Cohorts on the Basis of the Design or Analysis** | **1) Assessment of Outcome** | **2) Was Follow-Up Long Enough for Outcomes to Occur** | **3) Adequacy of Follow Up of Cohorts** |  |
| 1 | Wu et al. 2022 [12] | 1 | 1 | 1 | 1 | 2 | 1 | 1 | 0 | 8 |
| 2 | Pan et al. 2025 [22] | 1 | 1 | 1 | 1 | 2 | 1 | 1 | 0 | 8 |
| 3 | Calili et al. 2025 [19] | 1 | 1 | 1 | 0 | 2 | 1 | 0 | 1 | 7 |
| 4 | Baek et al. 2024 [18] | 1 | 1 | 1 | 1 | 2 | 1 | 0 | 1 | 8 |
| 5 | Jeong et al. 2018 [4] | 1 | 1 | 1 | 1 | 1 | 1 | 1 | 1 | 8 |
| 6 | Wełna et al. 2023 [6] | 1 | 1 | 1 | 1 | 1 | 1 | 1 | 1 | 8 |
| 7 | Xie et al. 2025 [5] | 1 | 1 | 1 | 1 | 2 | 1 | 1 | 1 | 9 |
| 8 | Wang et al. 2022 [21] | 1 | 1 | 1 | 1 | 1 | 1 | 1 | 1 | 8 |
| 9 | Li et al. 2023 [20] | 1 | 1 | 1 | 1 | 2 | 1 | 1 | 0 | 8 |
| 10 | Cai et al. 2025 [10] | 1 | 1 | 1 | 1 | 2 | 1 | 1 | 1 | 9 |
| 11 | Wang et al. 2023 [23] | 1 | 1 | 1 | 1 | 2 | 1 | 1 | 1 | 9 |

**Table S2. Search Strategy (PubMed)**

| #1 | **(Sepsis[MeSH Terms])** OR (Sepsis[Title/Abstract] OR Bloodstream Infection*[Title/Abstract] OR Septicemia*[Title/Abstract] OR Blood Poisoning*[Title/Abstract] OR Severe Sepsis[Title/Abstract] OR Pyemia*[Title/Abstract] OR Pyaemia*[Title/Abstract] OR Pyohemia*[Title/Abstract] OR abdominal sepsis[Title/Abstract] OR focal sepsis[Title/Abstract] OR intraabdominal sepsis[Title/Abstract] OR sepsis syndrome[Title/Abstract] OR septic disease[Title/Abstract]) | 252045 |
| --- | --- | --- |
| #2 | **(Nutrition Assessment[MeSH Terms])** OR (Nutrition Assessment[Title/Abstract] OR Nutritional Assessment*[Title/Abstract] OR Nutrition Index*[Title/Abstract] OR Nutrition Indices[Title/Abstract] OR Nutritional Index[Title/Abstract] OR Nutritional Indices[Title/Abstract] OR PNI[Title/Abstract] OR Prognostic Nutritional Index[Title/Abstract] OR Prognostic Nutritional Indices[Title/Abstract] OR Mini Nutritional Assessment*[Title/Abstract] OR Mini Nutrition Assessment*[Title/Abstract] OR nutritional screening[Title/Abstract] OR nutritional score[Title/Abstract] OR Controlling Nutritional Status[Title/Abstract] OR CONUT[Title/Abstract] OR Nutritional Risk Screening 2002[Title/Abstract] OR NRS 2002[Title/Abstract] OR Nutrition Risk in the Critically Ill[Title/Abstract] OR NUTRIC[Title/Abstract] OR modified Nutrition Risk in the Critically Ill[Title/Abstract] OR mNUTRIC[Title/Abstract] OR Mini Nutritional Assessment[Title/Abstract] OR MNA[Title/Abstract] OR Subjective Global Assessment[Title/Abstract] OR SGA[Title/Abstract] OR Malnutrition Universal Screening Tool[Title/Abstract] OR MUST[Title/Abstract] OR modified Glasgow Prognostic Score[Title/Abstract] OR mGPS[Title/Abstract] OR BUN-to-albumin ratio[Title/Abstract] OR BAR[Title/Abstract] OR dietary assessment[Title/Abstract] OR dietary evaluation[Title/Abstract] OR nutrition assessment[Title/Abstract] OR nutritional evaluation[Title/Abstract] OR nutritional assessment[Title/Abstract] OR geriatric nutritional risk index[Title/Abstract]) | 89073 |
| #3 | **(Prognosis[MeSH Terms])** OR (prognosis[Title/Abstract] OR Prognoses[Title/Abstract] OR Prognostic Factor*[Title/Abstract] OR prediction[Title/Abstract] OR mortality[Title/Abstract] OR survival analysis[Title/Abstract] OR prognostic[Title/Abstract] OR predictive value[Title/Abstract]) | 3975183 |
| #4 | (**(cohort studies[MeSH Terms])** OR **(Case-Control Studies[MeSH Terms])**) OR (Cohort Stud*[Title/Abstract] OR Concurrent Stud*[Title/Abstract] OR Closed Cohort Stud*[Title/Abstract] OR Historical Cohort Stud*[Title/Abstract] OR Incidence Stud*[Title/Abstract] OR Cohort Analys*[Title/Abstract] OR Birth Cohort Stud*[Title/Abstract] OR cohort fertility[Title/Abstract] OR cohort life cycle[Title/Abstract] OR Case-Control Stud*[Title/Abstract] OR Case-Comparison Stud*[Title/Abstract] OR Case-Compeer Studies[Title/Abstract] OR Case Base Studies[Title/Abstract] OR Case Referent Stud*[Title/Abstract] OR Matched Case Control Stud*[Title/Abstract] OR Nested Case-Control Stud*[Title/Abstract] OR matched case control[Title/Abstract]) | 3255607 |
| #5 | #1 AND #2 AND #3 AND #4 | 188 |

**Table S3. Search Strategy (Embase)**

| #1 | 'sepsis'/exp | 407,388 |
| --- | --- | --- |
| #2 | 'sepsis':ab,kw,ti OR 'bloodstream infection*':ab,kw,ti OR 'septicemia*':ab,kw,ti OR 'blood poisoning*':ab,kw,ti OR 'severe sepsis':ab,kw,ti OR 'pyemia*':ab,kw,ti OR 'pyaemia*':ab,kw,ti OR 'pyohemia*':ab,kw,ti OR 'abdominal sepsis':ab,kw,ti OR 'focal sepsis':ab,kw,ti OR 'intraabdominal sepsis':ab,kw,ti OR 'sepsis syndrome':ab,kw,ti OR 'septic disease':ab,kw,ti | 270,079 |
| #3 | 'nutritional assessment'/exp | 47,612 |
| #4 | 'nutrition assessments':ab,kw,ti OR 'nutritional assessment*':ab,kw,ti OR 'nutrition index*':ab,kw,ti OR 'nutrition indices':ab,kw,ti OR 'nutritional index':ab,kw,ti OR 'nutritional indices':ab,kw,ti OR 'pni':ab,kw,ti OR 'prognostic nutritional index':ab,kw,ti OR 'prognostic nutritional indices':ab,kw,ti OR 'mini nutritional assessment*':ab,kw,ti OR 'mini nutrition assessment*':ab,kw,ti OR 'nutritional screening':ab,kw,ti OR 'nutritional score':ab,kw,ti OR 'controlling nutritional status':ab,kw,ti OR 'nutritional risk screening 2002':ab,kw,ti OR 'nrs 2002':ab,kw,ti OR 'nutrition risk in the critically ill':ab,kw,ti OR 'modified nutrition risk in the critically ill':ab,kw,ti OR 'mini nutritional assessment':ab,kw,ti OR 'subjective global assessment':ab,kw,ti OR 'malnutrition universal screening tool':ab,kw,ti OR 'modified glasgow prognostic score':ab,kw,ti OR 'bun-to-albumin ratio':ab,kw,ti OR 'bar':ab,kw,ti OR 'dietary assessment':ab,kw,ti OR 'dietary evaluation':ab,kw,ti OR 'nutrition assessment':ab,kw,ti OR 'nutritional evaluation':ab,kw,ti OR 'nutritional assessment':ab,kw,ti OR 'geriatric nutritional risk index':ab,kw,ti | 933,929 |
| #5 | 'prognosis'/exp | 1,039,971 |
| #6 | 'prognosis':ti,ab,kw OR 'prognoses':ti,ab,kw OR 'prognostic factor*':ti,ab,kw OR 'prediction':ti,ab,kw OR 'mortality':ti,ab,kw OR 'survival analysis':ti,ab,kw OR 'prognostic':ti,ab,kw OR 'predictive value':ti,ab,kw | 3,684,053 |
| #7 | 'cohort analysis'/exp | 1,495,950 |
| #8 | 'case control study'/exp | 264,421 |
| #9 | 'cohort stud*':ab,kw,ti OR 'concurrent stud*':ab,kw,ti OR 'closed cohort stud*':ab,kw,ti OR 'historical cohort stud*':ab,kw,ti OR 'incidence stud*':ab,kw,ti OR 'cohort analys*':ab,kw,ti OR 'birth cohort stud*':ab,kw,ti OR 'cohort fertility':ab,kw,ti OR 'cohort life cycle':ab,kw,ti OR 'case-control stud*':ab,kw,ti OR 'case-comparison stud*':ab,kw,ti OR 'case-compeer studies':ab,kw,ti OR 'case base studies':ab,kw,ti OR 'case referent stud*':ab,kw,ti OR 'matched case control stud*':ab,kw,ti OR 'nested case-control stud*':ab,kw,ti OR 'matched case control':ab,kw,ti | 844,963 |
| #10 | #1 OR #2 | 476,685 |
| #11 | #3 OR #4 | 963,232 |
| #12 | #5 OR #6 | 3,921,855 |
| #13 | #7 OR #8 OR #9 | 1,880,744 |
| #14 | #10 AND #11 AND #12 AND #13 | 604 |

**Table S4. Search Strategy (Cochrane)**

| #1 | MeSH descriptor: [Sepsis] | 6664 |
| --- | --- | --- |
| #2 | (Sepsis):ti,ab,kw OR (Bloodstream Infection*):ti,ab,kw OR (Septicemia*):ti,ab,kw OR (Blood Poisoning*):ti,ab,kw OR (Severe Sepsis):ti,ab,kw OR (Pyemia*):ti,ab,kw OR (Pyaemia*):ti,ab,kw OR (Pyohemia*):ti,ab,kw OR (abdominal sepsis):ti,ab,kw OR (focal sepsis):ti,ab,kw OR (intraabdominal sepsis):ti,ab,kw OR (sepsis syndrome):ti,ab,kw OR (septic disease):ti,ab,kw | 18510 |
| #3 | #1 OR #2 | 20974 |
| #4 | MeSH descriptor: [Nutrition Assessment] | 983 |
| #5 | (Nutrition Assessments):ti,ab,kw OR (Nutritional Assessment*):ti,ab,kw OR (Nutrition Index*):ti,ab,kw OR (Nutrition Indices):ti,ab,kw OR (Nutritional Index):ti,ab,kw OR (Nutritional Indices):ti,ab,kw OR (PNI):ti,ab,kw OR (Prognostic Nutritional Index):ti,ab,kw OR (Prognostic Nutritional Indices):ti,ab,kw OR (Mini Nutritional Assessment*):ti,ab,kw OR (Mini Nutrition Assessment*):ti,ab,kw OR (nutritional screening):ti,ab,kw OR (nutritional score):ti,ab,kw OR (Controlling Nutritional Status):ti,ab,kw OR (CONUT):ti,ab,kw OR (Nutritional Risk Screening 2002):ti,ab,kw OR (NRS 2002):ti,ab,kw OR (Nutrition Risk in the Critically Ill):ti,ab,kw OR (NUTRIC):ti,ab,kw OR (modified Nutrition Risk in the Critically Ill):ti,ab,kw OR (mNUTRIC):ti,ab,kw OR (Mini Nutritional Assessment):ti,ab,kw OR (MNA):ti,ab,kw OR (Subjective Global Assessment):ti,ab,kw OR (SGA):ti,ab,kw OR (Malnutrition Universal Screening Tool):ti,ab,kw OR (MUST):ti,ab,kw OR (modified Glasgow Prognostic Score):ti,ab,kw OR (mGPS):ti,ab,kw OR (BUN-to-albumin ratio):ti,ab,kw OR (BAR):ti,ab,kw OR (dietary assessment):ti,ab,kw OR (dietary evaluation):ti,ab,kw OR (nutrition assessment):ti,ab,kw OR (nutritional evaluation):ti,ab,kw OR (nutritional assessment):ti,ab,kw OR (geriatric nutritional risk index):ti,ab,kw | 93101 |
| #6 | #4 OR #5 | 93101 |
| #7 | MeSH descriptor: [Prognosis] | 231209 |
| #8 | (prognosis):ti,ab,kw OR (Prognoses):ti,ab,kw OR (Prognostic Factor*):ti,ab,kw OR (prediction):ti,ab,kw OR (mortality):ti,ab,kw OR (survival analysis):ti,ab,kw OR (prognostic):ti,ab,kw OR (predictive value):ti,ab,kw | 237200 |
| #9 | #7 OR #8 | 406424 |
| #10 | MeSH descriptor: [Cohort Studies] | 211449 |
| #11 | MeSH descriptor: [Case-Control Studies] | 29781 |
| #12 | (Cohort Stud*):ti,ab,kw OR (Concurrent Stud*):ti,ab,kw OR (Closed Cohort Stud*):ti,ab,kw OR (Historical Cohort Stud*):ti,ab,kw OR (Incidence Stud*):ti,ab,kw OR (Cohort Analys*):ti,ab,kw OR (Birth Cohort Stud*):ti,ab,kw OR (cohort fertility):ti,ab,kw OR (cohort life cycle):ti,ab,kw OR (Case-Control Stud*):ti,ab,kw OR (Case-Comparison Stud*):ti,ab,kw OR (Case-Compeer Studies):ti,ab,kw OR (Case Base Studies):ti,ab,kw OR (Case Referent Stud*):ti,ab,kw OR (Matched Case Control Stud*):ti,ab,kw OR (Nested Case-Control Stud*):ti,ab,kw OR (matched case control):ti,ab,kw | 239438 |
| #13 | #10 OR #11 OR #12 | 399197 |
| #14 | #3 AND #6 AND #9 AND #13 | 238 |

**Table S5. Search Strategy (Web of Science)**

| #1 | Sepsis OR Bloodstream Infection* OR Septicemia* OR Blood Poisoning* OR Severe Sepsis OR Pyemia* OR Pyaemia* OR Pyohemia* OR abdominal sepsis OR focal sepsis OR intraabdominal sepsis OR sepsis syndrome OR septic disease (Topic) | 151996 |
| --- | --- | --- |
| #2 | Nutrition Assessment OR Nutritional Assessment* OR Nutrition Index* OR Nutrition Indices OR Nutritional Index OR Nutritional Indices OR PNI OR Prognostic Nutritional Index OR Prognostic Nutritional Indices OR Mini Nutritional Assessment* OR Mini Nutrition Assessment* OR nutritional screening OR nutritional score OR Controlling Nutritional Status OR CONUT OR Nutritional Risk Screening 2002 OR NRS 2002 OR Nutrition Risk in the Critically Ill OR NUTRIC OR modified Nutrition Risk in the Critically Ill OR mNUTRIC OR Mini Nutritional Assessment OR MNA OR Subjective Global Assessment OR SGA OR Malnutrition Universal Screening Tool OR MUST OR modified Glasgow Prognostic Score OR mGPS OR BUN-to-albumin ratio OR BAR OR dietary assessment OR dietary evaluation OR nutrition assessment OR nutritional evaluation OR nutritional assessment OR geriatric nutritional risk index (Topic) | 1217243 |
| #3 | TS=(prognosis OR Prognoses OR Prognostic Factor* OR prediction OR mortality OR survival analysis OR prognostic OR predictive value) | 3742046 |
| #4 | Cohort Stud* OR Concurrent Stud* OR Closed Cohort Stud* OR Historical Cohort Stud* OR Incidence Stud* OR Cohort Analys* OR Birth Cohort Stud* OR cohort fertility OR cohort life cycle OR Case-Control Stud* OR Case-Comparison Stud* OR Case-Compeer Studies OR Case Base Studies OR Case Referent Stud* OR Matched Case Control Stud* OR Nested Case-Control Stud* OR matched case control (Topic) | 2270897 |
| #5 | #1 AND #2 AND #3 AND #4 | 550 |
